# Use of physical restraints in nursing homes: a multicentre cross-sectional study

**DOI:** 10.1186/s12877-015-0125-x

**Published:** 2015-10-21

**Authors:** Hedi Hofmann, Ewald Schorro, Burkhard Haastert, Gabriele Meyer

**Affiliations:** Department of Health, University of Applied Sciences, Rosenbergstrasse 59, P.O. Box, CH-9001 St.Gallen, Switzerland; Department of Nursing Science, Witten/Herdecke University, Witten, Germany; School of Health, University of Applied Sciences and Arts Western Switzerland, Fribourg, Switzerland; mediStatistica, Neuenrade, Germany; Institute of Health and Nursing Science, Medical Faculty, Martin Luther University Halle-Wittenberg, Halle (Saale), Germany

**Keywords:** Nursing, Physical restraint, Nursing homes, Epidemiology, Prevalence

## Abstract

**Background:**

Although many countries have implemented strict legal rules, the prevalence of physical restraints in nursing homes seems to remain high. In Switzerland, data related to the frequency of physical restraints are scarce and little is known about associations with resident and nursing home characteristics. The aim of this study was to investigate the prevalence and types of physical restraints in nursing homes in two Swiss cantons and to explore whether resident-related and organisational factors are associated with the use of physical restraints.

**Methods:**

We conducted a multicentre cross-sectional study. Twenty nursing homes with 1362 residents from two culturally different cantons were included. Data on physical restraints and residents’ characteristics were extracted from residents’ records (11/2013 to 2/2014). Organisational data were collected by questionnaires addressing nursing home directors or nursing managers. Sample size calculation and outcome analysis took cluster-adjustment into account. Descriptive statistics and multiple logistic regression analysis with nursing homes as random effect were used for investigation.

**Results:**

The prevalence of residents with at least one physical restraint was 26.8 % (95 % confidence interval [CI] 19.8–33.8). Centre prevalence ranged from 2.6 to 61.2 %. Bilateral bedrails were most frequently used (20.3 %, 95 % CI 13.5–27.1). Length of residence, degrees of care dependency and mobility limitation were significantly positively associated with the use of physical restraint, but none of the organisational characteristics was significantly associated.

**Conclusion:**

Approximately a quarter of the nursing home residents included in our study experienced physical restraints. Since variation between nursing homes was pronounced, it seems to be worthwhile to explore nursing homes with particularly low and high use of physical restraints in future research, especially by using qualitative methods. There is a need for effective interventions aiming at restraint-free nursing care. Development of interventional approaches should consider specific residents’ characteristics associated with restraint use.

## Background

The use of physical restraints in nursing homes has been the subject of numerous empirical studies and discussion papers during recent years. Even though there are strict legal regulations in many countries, the prevalence of physical restraints in nursing homes seems still to be high [1, 2]. Due to methodological reasons and different definitions of physical restraints [3], a wide variation in the reported prevalence could be found. However, cross-national data by Feng et al. [4], using standardised methods for data collection in five countries, suggested a range of prevalence from 6 to 31 %. In Switzerland, data were collected in 94 nursing homes and revealed a prevalence of 6 %. Bedrails were excluded and all five participating cantons (i.e. member states of the Swiss Confederation) belonged to the German-speaking part of the country [4]. In another Swiss study data were collected on various quality indicators in nursing homes and a bedrail prevalence of 18.5 % was found [5]. Studies conducted in Germany [2, 6] reported prevalence figures of approximately 30 %. Variation of physical restraint prevalence between nursing homes seems to be pronounced, as indicated by Meyer et al. [6], reporting a range between 4 and 59 % residents with at least one physical restraint. According to a widely accepted definition, we understand physical restraint as ‘any device, material or equipment attached to or near a person’s body and which cannot be controlled or easily removed by the person and which deliberately prevents or is deliberately intended to prevent a person’s free body movement to a position of choice and/or a person’s normal access to their body’ [7].

From an ethical perspective, the use of physical restraints is highly questionable since the devices constrict residents’ dignity and autonomy and thereby harm personal integrity [8]. Decisions about the use of physical restraints in the care of older persons are complex and influenced by different factors like attitudes of health care staff, characteristics of residents as well as nursing home and environmental characteristics [9]. Even when nurses have negative feelings towards physical restraints, they identify a need for their use in specific situations and for various reasons like fall prevention or control of challenging behaviour [10]. The risk of using physical restraints is increased in residents with a low level of activities of daily living (ADL). Low cognitive function, repeated verbal and physical agitation as well as severe mobility impairments, previous accidental falls and related fractures are further factors associated with the use of physical restraints [3, 11]. In nursing homes with dementia-specific care units, special quality improvement strategies and public ownership, physical restraints seem to be used less often [9, 12, 13]. Staffing level seems not to be associated with the use of physical restraints [9, 11].

Physical restraints are associated with various negative outcomes like reduced residents’ quality of life, worse physiological and psychological condition [1], decrease of physical activity level, reduction of cognitive functioning, worsening of dementia-related behavioural symptoms [14] as well as increase of accidental falls, pressure ulcers and incontinence [3]. The majority of serious direct injuries from bedrails are caused by incorrect application or use of non-appropriate devices. Evidence on health benefits through the application of physical restraints, such as prevention of injuries, are lacking [15].

Various interventions to reduce physical restraints in nursing homes have been developed [16]. Recently published studies indicate the effectiveness of multifactorial preventive approaches. Physical restraints were reduced [17] and the use of belts decreased significantly [18, 19], also in newly admitted residents [20].

In Switzerland, according to a new law valid since January 2013, the use of physical restraints is allowed under exceptional circumstances only for residents who are at risk of harming themselves or others [21]. Data concerning the frequency of physical restraint use in Swiss nursing homes are scarce and little is known especially about the application of bedrails. Country-based prevalence data are required for a better understanding of the practice of physical restraint use and for decision-making about the need for interventions to reduce physical restraints. In the present study, we aimed to determine how often all types of physical restraints are applied among nursing home residents in two Swiss cantons, St.Gallen and Fribourg, and whether specific characteristics of residents and nursing homes are associated with a higher likelihood of restraint use. According to the federal structure of Switzerland, a dominant part of the health care system is regulated at the cantonal level, particularly in the case of nursing homes. Nursing practice in the cantons is also influenced by the respective language culture. There is evidence that differences in regulation and common practice may result in differences of physical restraint use [22]. St.Gallen belongs to German-speaking Switzerland and Fribourg to French-speaking Switzerland (Romandy) closely associated with French culture and the Roman as well as Latin tradition.

## Methods

### Design

We conducted a multicentre cross-sectional study.

### Sample/Participants

One hundred and eight nursing homes with at least 30 beds were eligible for inclusion. In a first step we built strata considering the characteristics “canton”, “size of nursing home”, and “rural or urban environment”. From these strata, nursing homes were randomly selected and consecutively invited until 20 institutions with a total of 1362 residents agreed to participate. Twelve institutions denied participation due to lack of time, nursing shortage, refurbishment and lack of interest. In total, we sent 32 invitation letters and gave 21 personal presentations about the study. Recruitment took place from July 2013 to January 2014. In each nursing home, a responsible nurse for the coordination of the study was nominated.

Based on Feng et al. [4] and Meyer et al. [6] the mean prevalence of physical restraints (including bedrails) was assumed to be 25 %. Experts and nursing home directors (*n* = 5) in Switzerland judged this figure as realistic. Without cluster adjustment a sample size of 214 would have been sufficient to reach a 95 % confidence interval of 25 % ± 6 % = 19–31 %. For proper cluster adjustment, we applied an intracluster correlation coefficient (ICCC) of 0.08, according to Meyer et al. [6]. Assuming a mean cluster size (=number of participants per home) of 68, a design factor of DF = 1 + (m-1) × ICCC [m = mean cluster size] = 1 + (68–1) × 0.08 = 6.36 is concluded [23]. Distributing 6.36 * 214 = 1361 residents over homes with mean cluster sizes of 68 overall 20 homes are needed.

### Data collection

Data collection was performed between November 2013 and February 2014. Data were obtained by trained external investigators (HH, ES, two study assistants) using the web-based software SecuTrial®.

Data collection on physical restraints covered items taken from the Maastricht Attitude Questionnaire (MAQ) [24, 25] as bilateral bedrails, unilateral bedrail at one side of the bed with the other positioned at the wall, belt in bed, sleep suit, tight sheet (a sheet over belly and upper legs that is tightened firmly under the mattress at both sides of the bed), wrist restraint, ankle restraint, chair preventing rising (deep or overturned chair), chair with a locked tray table, wheelchair with a locked tray table and belt in chair. The nominated nurses gathered data from the residents’ BESA or RAI records [26, 27] and nursing reports. In Switzerland, BESA (in German: Bedarfsklärungs- und Abrechnungs-System, in English: Needs Assessment and Payoff System) [26] and RAI (Resident Assessment Instrument) [27] are two established standard tools to assess residents’ care needs and resources and to document nursing services. According to the Swiss federal law on the reorganization of care funding [28], twelve different degrees of care dependency can be distinguished. Degree one is related to the lowest care dependency and degree twelve to the highest. BESA and RAI data have to be updated every six month since nurses in Switzerland are obliged to regularly assess residents’ conditions and needs. Data considering age, gender, degree of care dependency, length of stay in the nursing home, mobility restrictions, accidental falls, and fall-related fractures were collected.

Data concerning nursing home characteristics covered ownership, number of beds, presence of dementia-specific care units, staffing ratio, skills-/grade-mix of the nursing team, in-house or external physicians, access control to the nursing home, availability of guidelines and documentation standards aimed to control the use of physical restraints. These data were collected by questionnaires addressing nursing home directors or nursing managers of the 20 participating institutions.

### Ethical considerations

The ethics committee of the Canton of St.Gallen and the ethics committee for research of the Canton of Fribourg approved the study protocol. The approval covered all participating centers. The external investigators had neither direct access to the residents’ records nor personal contact with the residents. Residents were not identified by name during data collection and analysis. Thus, data protection was fully guaranteed. According to the ethics committees, no additional informed consent was required from the nursing home directors completing the questionnaires.

### Data analysis

Baseline characteristics of nursing homes and residents were expressed as means ± standard deviations (SD), numbers, and percentages. The primary outcome of the study was prevalence of restraints (resident with at least one physical restraint). All parameters describing the use of physical restraints as well as resident-related and organisation-related factors were considered as outcomes. These outcome variables are correlated within the clusters, such that simple variance estimators are biased (too low). Variances and confidence intervals for prevalence were adjusted for cluster correlation using methods described by Donner and Klar [23]. In case of unequal cluster sizes (cluster sizes vary between 36 and 164) the use of minimum variance weights was recommended by Kerry and Bland [29] because of a smaller increase of the variance due to the design effect from clustering. For the outcome variables, the cluster-correlation was estimated by the corresponding intra-cluster correlation coefficient (ICCC) and the design factor DF which depends on ICCC, cluster structure and weights. Finally, cluster-adjusted approximate two-sided 95 % confidence intervals (CI) for prevalence were calculated.

Associations between resident-related and organisation-related factors and physical restraint use were investigated by generalized linear mixed (logistic) models [30]. The binary variable “restraint yes/no” was the dependent variable and baseline variables were the independent variables as fixed effects. Cluster-adjustment was achieved by including clusters as random effects. In these models cluster-adjusted odds ratios (AORs) were estimated.

A set of baseline variables (Table 4) on nursing homes and residents’ characteristics were evaluated in cluster-adjusted univariate models (i.e. for each variable one separate model). Additionally, one big multiple model was fitted, including all variables. All significant variables from the univariate models and the big multiple model were selected for a final model. Significant variables from univariate models not being significant in the big multiple model were excluded. Furthermore, data concerning sex, age and agitation were selected for epidemiological or clinical reasons.

The level of significance was 5 %. All tests and confidence intervals were two-sided. Statistical analysis was performed using SPSS 21 and SAS 9.4.

### Rigour

To ensure completeness and correctness, a random sample of 20 % of the data were independently checked by two investigators. The absolute agreement of this check was 96.3 %. The entire data set was checked on plausibility in a systematic procedure by the biometrician of the study (BH).

## Results

Baseline characteristics of nursing homes and residents are displayed in Tables 1 and 2. Two third of the nursing homes were located in a rural area, 70 % had a non-profit ownership and the average number of beds per home was *n* = 70. Residents’ age ranged from 42 to 105 years, with a mean age of 85.1 (SD 8.4) and the median age of 86.0; 71.7 % of residents were female. Approximately 70 % of the residents were allocated to the degrees of care dependency four to nine, indicating a medium to high level of care dependency.

**Table 1:** Baseline characteristics of nursing homes (*n* = 20)

Characteristics	
Region	
St.Gallen	12 (60)
Fribourg	8 (40)
Location	
Urban	7 (35)
Rural	13 (65)
Ownership	
Non-profit	14 (70)
Private	5 (25)
Other	1 (5)
Nursing homes with ≥ 1 dementia care unit	7 (35)
Residents per nursing home	68.1 ± 30.4 (36–164)
Residents per caregiver FTE^a^	1.7 ± 0.4 (1.1–2.7)
Residents per night nurse	30.9 ± 8.0 (18.3–48.5)
Proportion of trained nursing staff	44.6 ± 9.3 (33.6–65.9)
≥1 nurse educated in psychogeriatrics	16 (80)
In-house education on the use of physical restraints during preceding 24 months	9 (45)
Medical care	
In-house physicians	2 (10)
Visiting general practitioners	9 (45)
In-house and visiting general practitioners	9 (45)
Specific documentation sheet for physical restraint	19 (95)
In-house standard for physical restraints	12 (60)
With definition of physical restraints^b^	9 (75)
Access control to the nursing home	16 (80)
Reception area at the entrance	15 (75)
Video surveillance	2 (10)
Light barrier at the entrance	3 (15)
Alarm system	6 (30)
Ward access by code or key	5 (25)

Values are numbers (percentage) or mean ± standard deviation (range). ^a^ *FTE* full time equivalent; ^b^percentage from 12 homes with in-house standard

**Table 2:** Baseline characteristics of nursing homes residents^a^(*n* = 1362)

Characteristics	
Region	
St.Gallen	844 (62)
Fribourg	518 (38)
Women	977 (71.7)
Age, years, mean ± SD (range)	85.1 ± 8.4 (42–105)
Length of residence, years, range (lower quartile, median, upper quartile)	0–60.4 (1.0, 2.3, 4.4)
Degree of care dependency	
1–3	351 (25.8)
4–6	408 (30.0)
7–9	518 (38.0)
10–12	85 (6.2)
Mobility limitation^b^	744 (56.2)
Verbal agitation^c^	303 (22.3)
Physical agitation^d^	172 (12.7)
Fall during preceding 30 days^e^	202 (14.8)
Fall during preceding > 30 to 180 days^e^	349 (25.6)
Hip fracture during preceding 180 days^f^	16 (1.2)
Other fracture during preceding 180 days^f^	25 (1.8)

Values are numbers (percentage) unless stated otherwise. ^a^Not cluster adjusted; ^b^ *n* = 38 missings; ^c^ *n* = 1 missing; ^d^ *n* = 2 missings; ^e^ *n* = 89 residents fulfilled both fall criteria and are counted twice; ^f^ *n* = 1 resident fulfilled both fracture criteria and was counted twice

### Prevalence and types of physical restraints

The cluster-adjusted prevalence of residents with at least one physical restraint was 26.8 % [95 % confidence interval (CI) 19.8–33.8], only slightly higher as expected. Centre prevalence ranged from 2.6 to 61.2 %. Bilateral bedrails and unilateral bedrails at one side of the bed with the other positioned at the wall were the most frequently used physical restraints [20.3 % (95 % CI 13.5–27.1) and 5.7 % (95 % CI 2.7–8.8)], followed by wheelchair with a locked tray table [1.8 % (95 % CI 0.6–3.1)], belt in chair [1.1 % (95 % CI 0.4–1.9)], and sleep suits [1.1 % (95 % CI 0.4–1.8)]. Further methods of physical restraints were scarcely or not reported at all (Table 3). Cluster adjusted prevalences in St.Gallen and Fribourg were 27.6 % (95 % CI 16.8–38.4) and 25.9 % (95 % CI 18.6–33.3 %), *p* = 0.993 (cf. univariate model in Table 4). The estimated intra-cluster correlation coefficients (ICCC) are presented in Table 3. The ICCC for the overall prevalence of 0.12 was higher than assumed in the sample size estimation, such that cluster adjustment results in larger variance estimations and CIs.

**Table 3:** Frequency of physical restraints

	Cluster-adjusted prevalence, % (95 % CI)	ICCC^a^	DF^b^
Residents with at least one physical restraint	26.8 (19.8–33.8)	0.1168	8.958
Bilateral bedrails	20.3 (13.5–27.1)	0.1319	9.973
Unilateral bedrail at one side of the bed with the other positioned at the wall	5.7 (2.7–8.8)	0.0847	6.805
Sleep suits	1.1 (0.4–1.8)	0.0092	1.684
Belt in bed	0.1 (0.0–0.3)	0.0066	1.496
Belt in chair	1.1 (0.4–1.9)	0.0133	1.969
Chair preventing rising	0.5 (0.0–1.2)	0.0190	2.366
Chair with a locked tray table	0.3 (0.0–0.6)	0.0005	1.039
Wheelchair with a locked tray table	1.8 (0.6–3.1)	0.0315	3.218

^a^ *ICCC* Intracluster correlation coefficient, ^b^ *DF* Design factor. Due to low prevalence of physical restraints (all outcomes except all physical restraints and bedrails on both sides) the asymptotical estimation of cluster-adjusted 95 % CI should be interpreted with caution

**Table 4:** Cluster-adjusted logistic regression models (cluster = random effect)

	Univariate models		Multiple model	
Variable	Odds ratio (95 % CI)	*p*-value	Odds ratio (95 % CI)	*p*-value
Nursing home characteristics				
Region, Fribourg vs. St.Gallen	1.00 (0.42–2.42)	0.993		
Location, rural vs. urban	0.92 (0.37–2.27)	0.848		
Ownership				
Private vs. non-profit	2.39 (0.98–5.83)	0.055		
Other vs. non-profit	3.36 (0.59–18.97)	0.155		
Dementia care unit, yes vs. no	1.13 (0.46–2.78)	0.773		
Residents per nursing home, ≤70 vs. >70	0.90 (0.37–2.20)	0.803		
Residents per caregiver FTE^a^ , ≤1.7 vs. > 1.7	2.39 (1.13–5.05)	0.025		
Residents per night nurse, ≤30 vs. > 30	1.76 (0.78–3.96)	0.159		
Proportion of trained nursing staff, ≤40 vs. >40	0.77 (0.32–1.83)	0.533		
Medical care				
General practitioner vs. in-house physician	0.39 (0.09–1.67)	0.187		
Others vs. in-house physician	0.52 (0.12–2.24)	0.354		
Physical restraint standard with definition, yes vs. no	1.08 (0.68–1.72)	0.735		
Resident characteristics				
Gender, men vs. women	1.00 (0.75–1.31)	0.975	1.27 (0.89–1.82)	0.189
Age				
75–84 vs. <75 years	0.76 (0.49–1.17)	0.213	0.87 (0.50–1.50)	0.606
85–94 vs. <75 years	0.92 (0.62–1.38)	0.701	1.27 (0.75–2.14)	0.369
≥ 95 vs. <75 years	1.14 (0.66–2.00)	0.634	1.49 (0.72–3.08)	0.283
Length of residence				
1.1–2.5 vs. 1 year	1.52 (1.04–2.22)	0.029	1.39 (0.88–2.19)	0.156
2.6–4.5 vs. 1 year	2.25 (1.53–3.31)	<0.001	1.44 (0.90–2.29)	0.127
4.6- vs. 1 year	3.18 (2.19–4.63)	<0.001	1.91 (1.22–3.02)	0.005
Degree of care dependency				
4–6 vs. <4	12.97 (5.02–33.51)	<0.001	9.93 (3.63–27.13)	<0.001
7–9 vs. <4	81.49 (32.02–207.40)	<0.001	50.06 (18.54–135.15)	<0.001
10–12 vs. <4	530.82 (183.67–1,534.09)	<0.001	294.25 (94.37–917.45)	<0.001
Mobility limitations, yes vs. no	7.55 (5.31–10.74)	<0.001	3.46 (2.35–5.10)	<0.001
Verbal agitation, yes vs. no	1.48 (1.11–1.98)	0.007	0.87 (0.58–1.29)	0.474
Physical agitation, yes vs. no	1.61 (1.11–2.32)	0.012	1.13 (0.68–1.86)	0.642
Fall				
Within the last 30 days, yes vs. no	0.98 (0.68–1.40)	0.902		
Between >30 and 180 days, yes vs. no	1.23 (0.91–1.65)	0.175		

Residents excluded from model due to missing values: Mobility limitations (*n* = 38), physical agitation (*n* = 1), verbal agitation (*n* = 2). ^a^ *FTE* full time equivalent

### Associations with the use of physical restraints

Table 4 displays the results of the cluster-adjusted univariate and multiple logistic regression analyses. Most significant univariate associations remain significant in the multiple model adjusted for the other variables with some changes of the estimated odds ratio. Only the positive association of the number of residents per caregiver [full time equivalent (FTE) (≤1.7 vs. >1.7)] in the univariate model (AOR 2.39, 95 % CI 1.14–5.05) was explained by the other variables in the big model containing all variables. In the final multiple model with selected variables, length of residence for more than 4.5 years (AOR 1.91, 95 % CI 1.22–3.02), degree of care dependency of 4–6 (AOR 9.93, 95 % CI 3.63–27.13), degree of care dependency of 7–9 (AOR 50.06, 95 % CI 18.54–135.15), degree of care dependency of 10–12 (AOR 294.25, 95 % CI 94.37–917.45) and mobility limitations (AOR 3.46, 95 % CI 2.35–5.10) are positively and significantly associated with the use of physical restraints. The variable “cognition” was not documented in a proper way in the BESA/RAI records and only few residents had either a diagnosis of dementia or a Mini-Mental State Examination. Therefore, data on cognitive status were incomplete and not suitable for inclusion into the analysis.

None of the organisational characteristics was significantly associated with a higher probability of physical restraint use.

## Discussion

Our cross-sectional study in two Swiss cantons indicates that nearly 27 of 100 residents were subject to at least one physical restraint. The pronounced variation of prevalence between nursing homes (2.6 % to 61.2 %) is in accordance with studies from Germany [6]. A recent European study with nursing home residents suffering from dementia in eight countries revealed a prevalence of physical restraints of 31.4 % with country variation ranging from 6.1 to 83.2 % [31]. Thus, a relation between nursing practice regarding physical restraints and cultural background is likely. Our study revealed no difference in the prevalence between the German-speaking and the French-speaking part of Switzerland. The enactment of the new Swiss-wide law in 2013 might have minimized any differences in the use of physical restraints. However, this is only speculation, since there is no previous prevalence study comparing two culturally different regions of Switzerland.

Bedrails were the most commonly used type of physical restraint, as reported by a recent study on nursing home quality in Switzerland [5]. The former study revealed a lower prevalence figure. Different data collection methods might have led to an underestimated prevalence. As described by earlier studies, nurses judge bilateral bedrails as a moderate restrictive measure and they do not hesitate to use them [24]. Therefore, one might assume that the application of physical restraints in some nursing homes is still routine care and used in order to prevent falls and ensure safety. However, bedrails do not always guarantee residents’ safety. On the contrary, as shown in other studies, they can also cause injuries and even death of residents by entrapment [32]. Limiting a resident’s movement by using chairs with a locked tray table was a rare type of physical restraint in our study. Belts in chairs or sleep suits were also seldom applied.

We found significant resident-related associations with the application of physical restraints, which were predominately bedrails. Increased degree of care dependency as well as mobility limitations revealed pronounced positive associations with restraint use. Earlier studies confirm these results [3, 5, 6]. Considering residents´ baseline characteristics, with 75 % of the residents displaying a higher degree of care dependency and more than half of the persons with mobility limitations, the probability of physical restraint is therefore notably elevated. An increased length of residence in the institution was also positively associated with the use of physical restraint. These results are confirmed by the cross-national study by Feng et al. [4] who described an association between an extended period of institutionalisation and the risk of restraint use in Switzerland. However, data from other participating countries (Canada, Hong Kong, Finland and USA) did not show any relation between longer resident stay and increased risk of physical restraint [4]. Our results do not indicate positive associations with gender or older age as described by former studies [11, 33]. Verbal and physical agitation was not associated with restraint use. As the baseline data show, these characteristics were mentioned rather rarely and are a surprising result, since recent studies indicated these characteristics as positively associated with restraint use [3]. However, this remains speculative, since comparisons with commonly used tools like the Neuropsychiatric Inventory (NPI) are lacking.

Only one organisational characteristic (residents per caregiver) turned out to be positively associated with physical restraint use in the univariate model. However, this association did not remain significant in the multiple model. Therefore, none of the organisational characteristics were significantly associated with physical restraint use. These results are in accordance with several earlier studies [2, 6, 34]. However, other studies indicate that physical restraints are less likely in dementia-specific care units [12, 35, 36] and in nursing homes with non-profit ownership [9, 37]. In general, the literature is inconsistent concerning associations of organisational factors with physical restraint use. Nevertheless, residents’ characteristics seem to have a greater influence on physical restraints use than organisational characteristics.

The persistent use of physical restraints in nursing homes requires effective interventions for educating nursing staff as well as all persons involved, e.g. residents, relatives, nursing experts and nursing homes directors. As recent studies have demonstrated, multicomponent interventions may reduce physical restraints in nursing homes [17, 19]. Since the results of our study are comparable to former studies from Germany [2, 6], the transfer and implementation of an effective intervention programme developed for nursing homes in Germany [17] might be appropriate.

With regard to the implementation of evidence-based practice, we have to take into consideration the role of nursing leadership. Effective leadership is vital in this process as well as the institution and the culture in which the leader operates [38]. Current evidence shows the relationship between positive relational leadership and lower physical restraint use [39]. Leaders can facilitate working conditions, create an atmosphere of open communication with staff and promote positive relationships among nurses in order to ensure safe, high-quality patient care and work engagement [40]. These components seem to be equally important to successfully reduce physical restraints.

### Strengths and limitations

One of the strengths of the current study is the inclusion of a large sample comprising 1362 residents from two culturally different regions of one country. The results provide the basis for better understanding of the practice of physical restraint use in Switzerland. However, we cannot exclude that the results are not exactly transferable to the other parts of the country. Our study contributes to the body of knowledge by adding new evidence from a sound association analysis of resident-related and organization-related characteristics. Personal visits to the nursing homes in order to explain in detail the performance of the study and the data collection ensured a trustworthy relationship with the nursing staff, which is a very important premise for research in an ethically sensitive area. The number of missing values was low (*n* = 41), which demonstrates an accurately performed data collection.

We did not collect the data by direct observation which is certainly the most valid method. However, as shown in validation studies, medical or residents’ records are a reliable source of information [41].

Former studies have shown that cognitive impairment is associated with the use of physical restraints [3]. Unfortunately, we could not explore a potential association since the data on cognitive status were not eligible for regression analysis.

## Conclusion

Our study results show substantial variation in the frequency of physical restraint use between nursing homes. This suggests that a reduction could be possible and the use of bedrails could be minimized in order to respect the persons’ autonomy and mobility. The new Swiss law may lead to more awareness with regard to physical restraints but further efforts towards restraint-free nursing care are required. Therefore, it seems to be worthwhile to explore the approach of nursing homes with particularly low and high use of physical restraints in future qualitative research. The development of interventions should consider specific residents’ characteristics associated with physical restraints.

Generally, the use of physical restraints must always consider the serious consequences for residents, their dignity and how their quality of life might be affected. The decision-making process concerning the application of physical restraints has always to respect the resident’s situation as well as nurse- and organisation-related factors. Leadership could essentially contribute to restraint-free nursing care by sensitizing nurses and creating optimal working conditions.

## Abbreviations

ADL: Activities of daily living
AOR: Adjusted odds ratio
BESA: Bedarfsklärungs- und Abrechnungs-System (in English: Needs Assessment and Payoff System)
CI: Confidence interval
DF: Design factor
FTE: Full time equivalent
ICCC: Intracluster correlation coefficient
MAQ: Maastricht attitude questionnaire
n: Number of characteristic values
NPI: Neuropsychiatric inventory
RAI: Resident assessment instrument
SAS: Statistical analysis systems
SD: Standard deviation
SPSS: Statistic package for social sciences
vs: Versus
